# Macrophage/microglia polarization for the treatment of diabetic retinopathy

**DOI:** 10.3389/fendo.2023.1276225

**Published:** 2023-09-28

**Authors:** Yujia Yao, Jiajun Li, Yunfan Zhou, Suyu Wang, Ziran Zhang, Qin Jiang, Keran Li

**Affiliations:** ^1^ Department of Ophthalmology, The Affiliated Eye Hospital of Nanjing Medical University, Nanjing, China; ^2^ The Fourth School of Clinical Medicine, Nanjing Medical University, Nanjing, China

**Keywords:** diabetic retinopathy, immune system, macrophages, microglia, ocular diseases, polarization

## Abstract

Macrophages/microglia are immune system defense and homeostatic cells that develop from bone marrow progenitor cells. According to the different phenotypes and immune responses of macrophages (Th1 and Th2), the two primary categories of polarized macrophages/microglia are those conventionally activated (M1) and alternatively activated (M2). Macrophage/microglial polarization is a key regulating factor in the development of inflammatory disorders, cancers, metabolic disturbances, and neural degeneration. Macrophage/microglial polarization is involved in inflammation, oxidative stress, pathological angiogenesis, and tissue healing processes in ocular diseases, particularly in diabetic retinopathy (DR). The functional phenotypes of macrophages/microglia affect disease progression and prognosis, and thus regulate the polarization or functional phenotype of microglia at different DR stages, which may offer new concepts for individualized therapy of DR. This review summarizes the involvement of macrophage/microglia polarization in physiological situations and in the pathological process of DR, and discusses the promising role of polarization in personalized treatment of DR.

## 1 Introduction

A crucial part of innate and acquired immunity is the immune cell class known as macrophages/microglia, which are produced by bone marrow progenitor cells. In response to certain stimuli from the ecological environment, macrophages undergo a process called macrophage polarization in which they develop distinct functional features (1). In different microenvironments macrophages/microglia may be phenotypically polarized to mount specific functional programs. M1 (classically activated macrophages) and M2 (alternatively activated macrophages) are the primary subcategories of polarized macrophages/microglia (2). Furthermore, polarized macrophages/microglia can affect local immunological responses in many ways (3–5). They are essential for the regulation of infectious and metabolic diseases, tumors, and neurodegenerative disorders (6–9).

In both healthy and unhealthy conditions, macrophages/microglia are crucial for physiological homeostasis. Physically, macrophages/microglia are highly tiled and ramified, and are characterized by a tiny soma with many branches and fine cellular processes. They incessantly, randomly, and repetitively expand and contract in all directions, serving as immunological surveillance. Phagocytosis, external antigen presentation, and management via cytokine and growth factor production are the main functions of macrophages and microglia in the pathological state. They can be activated by pattern recognition receptors to promote phagocytosis of cellular debris. In addition to their phagocytic functions, activated microglia can serve as antigen-presenting cells. They release pro-inflammatory cytokines and stimulate the immune system through direct cell-cell interaction. Furthermore, they upregulate induced nitric oxide synthase (iNOS) upon activation, which increases local nitric oxide concentrations, damages peripheral neurons, and promotes local capillary leakage.

**Figure 1:**
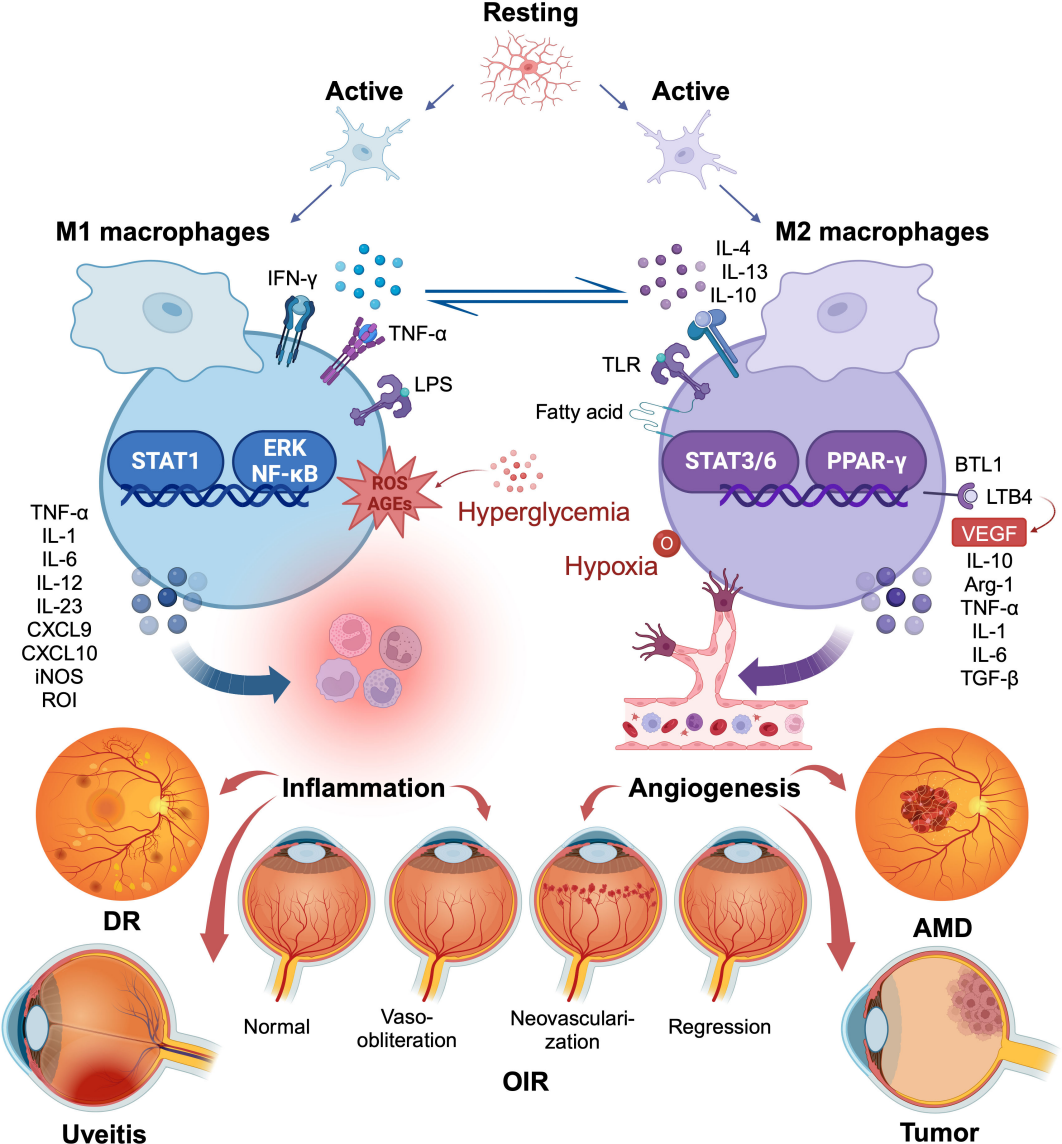
Macrophage/microglia polarization in several ocular disorders. The two polar opposites of the changed state of macrophage/microglia activation are M1 and M2. They are kinetically reversible; type M1 can shift to type M2 when microenvironmental conditions change, and vice versa. M1 macrophages produce pro-inflammatory chemicals that, although engulfing many pathogens, may also accelerate inflammatory processes that are detrimental. M2 macrophages, in contrast, lessen such harm, clear away necrotic cell debris, encourage tissue repair and remodeling, along with revascularization. The figure illustrates the common role of macrophage/microglia polarization in several ocular diseases. Created with BioRender.com. M1, conventionally activated macrophages; M2, alternatively activated macrophages.

Major causes of blindness, including diabetic retinopathy (DR), oxygen-induced retinopathy (OIR), age-related macular degeneration (AMD), uveitis, and tumors (uveal melanoma), are closely related to inflammation and immunity (10–13). The polarization of macrophages/microglia has recently been found to be associated with the onset of ocular diseases (14, 15). As a result of retinal ischemia and hypoxia, inflammatory cells such as macrophages and monocytes gather around the vessels (16). Macrophages are polarized in response to environmental stimuli. The majority of pro-angiogenic substances (including vascular endothelial growth factor [VEGF]) required for ocular neovascularization are secreted by these cells. Vascular endothelial cells and smooth muscle cells proliferate, differentiate, and migrate as a result of this process, bringing about the formation of harmed vessels and eventually retinal/vitreous hemorrhage (17). This association substantially influences the progression and prognosis of ocular diseases (**Figure 1**).

Diabetes mellitus is a metabolic disease featured with hyperglycemia caused by improper insulin production and insulin resistance. Microvascular complications occur most commonly in patients with DR and their prevalence is rapidly increasing worldwide (18). DR is subdivided into non-proliferative DR (NPDR) and proliferative diabetic retinopathy (PDR) decided by whether the retina undergoes neovascularization (19). The main factor that causes permanent blindness in working-age individuals is DR, particularly PDR (20). Damage to the neurovascular unit is currently considered an important pathogenic mechanism of diabetic retinopathy (21). Neurons, glial cells, basement membranes, pericytes, and endothelial cells comprise the retinal neurovascular unit (22). Increased neural activity results in greater blood flow to meet metabolic needs. Neuronal stress and apoptosis in the early stages of DR cause microglial activation and aggravate vascular damage. Vascular abnormalities further affect neuronal cell growth, metabolism, and function, making neural damage more severe. The neurovascular unit is gradually damaged, and eventually forms a microvascular lesion (23–25). A key player in the pathophysiology of DR is VEGF. Thus, anti-VEGF medications are currently the main DR treatment (26). Anti-VEGF medications are efficient in clinically inhibiting the development of neovascularization however they have no effect on angiogenic agents other than VEGF. Other treatment modalities, such as laser photocoagulation and vitrectomy, are often accompanied by significant side effects, including damage to healthy tissues, postoperative cataracts, and neuronal apoptosis (27–29). Therefore, new treatments for DR need to be developed.

In this review, the major duties of polarized microglia and macrophages in DR are examined, their research status and prospects for DR treatment are discussed.

## 2 Macrophage/microglia origin, classical activation, and functions

The current view is that during embryonic development, precursor cells from the yolk sac or embryo infiltrate organs and differentiate into tissue-resident macrophages (30, 31). Tissue-resident macrophages permanently survive and constantly self renew during adulthood and peripherally derived macrophages from monocytes also exist (32). Monocyte-derived macrophages mainly differentiate from blood monocytes produced from bone marrow hematopoietic stem cells. They are stimulated by multi-colony stimulating factor (CSF) and granulocyte-macrophage CSF to develop into monocytes, pre-monocytes, and mature monocytes. Subsequently, they spread throughout the epidermal and submucosal tissues of the body where they are vital for immunological surveillance, self-stabilization, and infection resistance (33). Macrophages can be further classified into microglia, osteoblasts, and within areas, such as lung macrophages, based on their anatomical location and function type. Microglia, a type of central nervous system tissue-resident macrophages, can engulf apoptotic neurons and trigger synaptic remodeling to maintain central nervous system homeostasis (34).

Macrophages and microglia are remarkably plastic, heterogeneous cells with multiple immunological functions. They are generally classified into two groups based on their function following activation: cc or M2 and caM or M1 type (35). However, continuous in-depth research on macrophage polarization indicates this process is constantly evolving. M1 and M2 are the two extreme states of macrophages, and the phenotypes of macrophages are altered under numerous normal and pathogenic conditions (1, 36).

M1 macrophages, whose surface markers include HLA-DR, CD80, CD86, and CD197, produce pro-inflammatory cytokines (37, 38). Typically, Th1 (interferon [IFN]-gamma, tumor necrosis factor-[TNF]-alpha) or bacterial lipopolysaccharide (LPS) can induce and activate M1 polarization, causing them to release large amounts of pro-inflammatory cytokines, including TNF-alpha, interleukin (IL)-1, IL-6, IL-12, IL-18, IL-23, and the chemokine ligands CXCL-9 and CXCL-10. They also generate significant amounts of iNOS, reactive oxygen intermediates (ROI), nitric oxide, and reactive oxygen species (ROS) (39, 40). Therefore, they are effector cells in the Th1 immune response, killing intracellular pathogens, removing foreign substances, and participating in the acute pro-inflammatory response (41–43). As a result, M1 macrophages may intensify inflammatory processes that are detrimental to tissue while eliminating pathogens.

M2 macrophages secrete anti-inflammatory molecules to mitigate this damage. They generate angiogenic mediators, including transforming growth factor (TGF-beta), VEGF, and epidermal growth factor, which reduce inflammation and accelerate wound healing. However, precisely because of the secretion of these factors and their overactivation, aberrant hyperplasia may result, such as pathological angiogenesis. Four distinct populations of M2 macrophages may be identified based on the multiple activation signals they receive: M2a, M2b, M2c, and M2d, whose surface markers include CD206, CD209, CD301, and CD163 (40, 44). Type II cytokines, IL-4 or IL-13, activate M2a macrophages, which then secrete several pro-inflammatory cytokines, including IL-10 and arginase 1, and receptors, such as mannose receptor CD206 and macrophage galactose type C lectin (MGL; CD301) (40). They are insensitive to inflammatory stimuli, however, they eliminate pathogens, remove debris, stimulate angiogenesis, and aid in tissue regeneration, thus promoting Th2 immunity. Immune complexes, toll-like receptors (TLR), IL-1R ligands, and LPS can trigger M2b macrophages. These cells secrete high levels of IL-10 and low levels of IL-12. In addition, they notably release several inflammatory cytokines such as TNF-alpha, IL-1, and IL-6, which reduce acute inflammation brought on by bacterial endotoxins and promote Th2 differentiation and humoral immunity (45). IL-10, TGF-beta, glucocorticoids, or open-loop steroid hormones (such as vitamin D) can activate M2c macrophages, also referred to as inactivated macrophages. They generate large amounts of TGF-beta and IL-10, which inhibit immunological inflammation (46).

M2d macrophages, also known as tumor-associated macrophages (TAM), are dominant in tumors. IL-6 and adenosine induce them to generate a lot of IL-10 and VEGF. Evidence suggests that macrophages can inhibit immunity, promote tumor cell proliferation, and stimulate angiogenesis (47, 48). TAM release more mannose receptor and scavenger receptor A than M1 macrophages, and fewer pro-inflammatory chemicals, such as IL-1beta, IL-6, IL-12, TNF-alpha, and ROI (47). Simultaneously, the levels of several macrophage differentiation markers in TAM, including F4/80, CD11b, CD68, and CD115, are lower than those in typical M2 macrophages. Therefore, more researches are of the opinion that TAM have traits common to both M1 and M2 polarization types and are in a stage of transition between them (49, 50). Thus, the ratio of the two polarization types is influenced by the tumor microenvironment, which promotes or inhibits tumor growth (51) (**Table 1**).

**Table 1:** Phenotypes, stimulations, secretions, and markers of M1 and M2 macrophages.

Phenotype	Stimulation	Secretion	Marker
M1	IFN-γ, LPS	TNF-α, IL-1, IL-6, IL-12, IL-18, IL-23	HLA-DR, CD80, CD86, CD197
M2a	IL-4, IL-13	IL-10, TGF-β, Arg-1	CD206, CD209, CD301, CD163
M2b	TLR, IL-1R, LPS	IL-1, IL-6, IL-10, TNF-α	
M2c	IL-10, TGF-β, glucocorticoids, open-loop steroid hormones	IL-10, TGF-β	
TAM	IL-6, adenosine	VEGF, IL-1β, IL-6, IL-10, IL-12, TNF-α	

IFN-γ, interferon‐gamma; IL, interleukin; LPS, lipopolysaccharide; M1, conventionally activated; M2, alternatively activated; TAM, tumor-associated macrophages; TGF-β, transforming growth factor-beta; TNF-α, tumor necrosis factor-alpha; TLR, toll-like receptors; VEGF, vascular endothelial growth factor.

Nevertheless, a disadvantage of this typing system is that it ignores macrophages undergoing positive division. Macrophage CSF-1 and IL-34 interact with the CSF receptor (CSF-1R) to induce macrophage proliferation and influence their differentiation, survival, and function (52). Studies have revealed that CSF-1-treated mouse microglia do not exhibit conventional M1 or M2 polarization, in contrast to earlier theories that macrophage CSF stimulation causes M2 polarization (53, 54). Therefore, the possibility of establishing M3 typing has been proposed (55). The diverse phenotypes and functions of macrophages enable them to play important roles in inflammation, pathogen resistance, tissue remodeling and repair, autoimmune regulation, and tumor suppression.

## 3 Retinal macrophages/microglia origin, distribution and functions

Microglia are macrophages assumed to be inherent in the central nervous system and fundamental for neuronal regeneration and immunological homeostasis (56). Primitive yolk sac macrophages are the source of microglia (57–59). In addition to tissue-resident microglia and monocyte-derived microglia in the bone marrow can penetrate the blood-retinal barrier under specific circumstances and show morphology and function similar to those of endogenous microglia in the retinal environment (60). After complete eradication of indigenous microglia in the retina, additional microglia recharge and originate from two different ways: macrophages in the ciliary body and iris, which arise peripherally (minority), and the optic nerve, which develop in the center (majority) (61, 62). This phenomenon confirms the presence of macrophage-derived microglia in the retinal periphery.

Microglia are mostly found in the inner layers of the retina in healthy individuals, including the nerve fiber layer (NFL), ganglion cell layer (GCL), inner plexiform layer (IPL), and outer plexiform layer (OPL) (25). At the age of 10-week-gestation, microglia have been found in the human retina (63). Moreover, their distribution is almost identical to that in mature retinas during development (64). Using immunocytochemistry and tomato lectin histochemistry, microglia are shown to be present in mouse retinas of 11.5-day-old embryos, distributed in the neuroblastic layer, IPL, and GCL just prior to birth, and with similar distribution as adult retinas from postnatal day 14 on (65). Microglia are also present in the OPL, ONL, IPL, GCL, and NFL of the primate retinas, resembling those of the human retinas (66). Microglia migrate to the retina in two ways. Before vascularization, most microglia migrate to the peripheral retina from nearby ocular tissues through the ciliary epithelium, primarily from the developing ciliary body and iris blood vessels. However, after vascularization, microglia originally derived from retinal blood vessels appear to migrate to the retina through the optic nerve head (67).

Conventional studies have assumed that microglia are dormant in healthy adult retinas and respond only when damaged. However, more recent studies indicate microglia have been found to be dynamic, constantly surveying the neural environment and performing tissue surveillance and intercellular communication functions (68–70). Using *in vitro* preparations, gradual and ongoing expansion and contraction of microglial processes may be observed, which suggest a key role in maintaining normal retinal function (71, 72). Anatomically, microglia are in direct contact with pericytes, which are strongly related to other components of the neurovascular unit nearby, to keep the normal function of the neurovascular unit and blood-retina barrier (BRB) integrity (73). Activated microglial cells are thought to play a crucial role in pathogenic situations, such as inflammation and traumatic and degenerative nerve injury. In response to damage, microglia upregulate process motility, reorient their shape and promote migratory activity (74). However, when frequently activated by alarm signals from both external and endogenous sources, microglial cells may become chronically hyperactive and secrete massive amounts of pro-inflammatory cytokines, which can result in an imbalance between the protective and injurious effects (75). Pericyte loss and endothelial cell damage result from the production of such inflammatory mediators (76). These mechanisms are present throughout DR development, and understanding the control of microglial polarization may lead to new perspectives for the development of individualized DR therapies.

## 4 Macrophage/microglia polarization in DR

### 4.1 Polarization in DR metabolism

Changes in blood glucose levels are a characteristic of diabetes mellitus. Altered glucose levels affect macrophage polarization and inflammatory cytokine secretion. Glucose transporter 1 has been reported to govern glycolysis, a process that is necessary for the generation of inflammatory cytokines and regulation of pro-inflammatory genes in microglia, indicating the participation of polarization in the pathological process (77). M1/M2 macrophage polarization is balanced in healthy human circulation. In contrast, deficiency in anti-inflammatory cells causes a significant reduction in M2 macrophage polarization, leading to an increase in M1/M2 polarization in the peripheral blood of patients with type 2 diabetes (78). Central obesity, insulin resistance, and abnormal glycemic control are typical characteristics of patients with type 2 diabetes and are all associated with a polarization imbalance. Chen et al. (79) obtained similar results. They observed that microglia stimulated by high glucose was initially polarized to the M2a phenotype, which protects against damaged tissues. However, over time, M1 pro-inflammatory factor production increases, M2 anti-inflammatory factor production decreases, and macrophages gradually tend to have the M2b phenotype (likely an intermediate phenotype between M2a and M1). In the late stage, microglia display an M1-like phenotype with pro-inflammatory effects. Furthermore, increased pro-inflammatory M1 monocyte-macrophages in the peripheral blood have been observed in individuals with preclinical diabetes, for instance impaired fasting glucose and/or impaired glucose tolerance patients (80). Similar findings were observed in the circulation of db/db mice, where pro-inflammatory cytokines (M1) and anti-inflammatory cytokines (M2) increased early (5 weeks), and by 20 weeks, db/db mice had considerably greater levels of pro-inflammatory cytokines compared to db/+ controls (81)..

Moreover, diabetes often coexists with hyperlipidemia, which is directly correlated with the onset of diabetic complications, such as DR, and accelerates progression (82). Research has shown that when TLR are activated by LPS, glucose absorption increases and glucose is oxidized to induce acetyl-CoA production, a precursor for the production of fatty acids. During inflammatory reactions, glucose metabolism is activated to promote *de novo* lipogenesis. Sterol regulatory element binding transcription factor 1a, which controls the production of inflammatory cytokines, is also expressed as a result of TLR activation (83). Compared to healthy people, patients with hypercholesterolemia had higher levels of pro-inflammatory CD68^(+)^ CCR2^(+)^ M1 monocyte macrophages, although anti-inflammatory CX3CR1^(+)^ CD163^(+)^/CD206^(+)^ M2 monocyte macrophages are decreased (84). Overall, a predominance of M1 macrophage polarization and increased secretion of pro-inflammatory cytokines have been observed in the peripheral blood of patients with diabetes.

According to recent studies, one of the most promising treatment options for diabetes may be to boost the polarization of the macrophage M2 phenotype to reduce insulin resistance and stabilize glucose/lipid metabolism (85–87). Additionally, this therapy may assist patients with diabetes in diminishing endothelial dysfunction and preventing complications of diabetes (88–90).

### 4.2 Microglia activation and polarization in animal models and patients with DR

An important characteristic of retinas with DR in both animals and humans is the migratory tendency of microglia. The retinal glia cells undergo alterations in both the early and late phases of DR development. A number of animal models have been created to comprehend the pathophysiology of DR. In DR model rats, M1 polarization increased, while M2 polarization decreased, and microglia were more prone to M1 polarization as the glucose concentration increased (91, 92). The iNOS (M1 marker) and arginase-1 (M2 marker) levels in the retinas of db/db mice were high at five-week-old; however, by eight-week-old, iNOS continued to rise, while arginase-1 returned to baseline levels. Retinal M1 and M2 proteins were also differentially distributed. M1-like proteins are mostly found in the outer segment, OPL, inner nuclear layer, and GCL, whereas M2-like proteins are primarily produced in the inner nuclear layer and GCL (81). Chen et al. (93) observed the activation and distribution of microglia in rats with hyperglycemia induced by Streptozotocin through OX-42 staining. The percentage of activated microglia considerably increased in the retinas of four-week-old rats with diabetes and remained high in eight- and twelve-week-old rats with diabetes. Twelve weeks following the initiation of diabetes, there appeared to be a redistribution of retinal microglia, with more in the NFL/GCL and fewer in the IPL, whereas there was no difference in the distribution of microglia in the NFL/GCL and IPL layers at 4 and 8 weeks. This demonstrates the motility of the microglia. The study by Omri et al. (94) seems to explain this phenomenon. In a Goto-Kakizaki rat model for spontaneous type 2 diabetes, the number of retinal pigment epithelium pores increased during the prophase of hyperglycemia (5 months). After long-term exposure to hyperglycemia (12 months), fewer retinal pigment epithelium pores remained, causing activated microglia/macrophages to accumulate subretinally and promote retinal injury (21, 94).

Similarly, clinical studies have demonstrated that microglia are significantly more prevalent in eyes with DR and display a hypertrophic pattern distinct from the ramified form during the resting state. The quantity of activated microglia, which are more common in the perivascular areas of the microaneurysms, slightly increases during NPDR. The number of activated microglia, which accumulate in cotton wool areas and the surrounding dilated arteries, significantly increases from NPDR to PDR. An abnormally high number of activated microglia develop in the GCL, surrounding newly formed juvenile arteries in the NFL and in the optic nerve head in PDR (95). These results imply that microglia in the retinas of DR patients are distributed similarly to 12-week-old diabetic mice. Thus, activated microglia are tightly associated with blood vessels that are closely connected geographically and promote microangiopathy in DR. Microglia/macrophages accumulate in the retina under the prolonged effects of hyperglycemia, indicating their contribution to the development of the disease. Optical coherence tomography angiography revealed that patients with PDR had significantly more macrophage-like cells surrounding their retinal vessels than healthy individuals, diabetics without DR, and patients with NPDR (96); diabetic macular edema was the most relevant factor contributing to the increase in macrophage-like cells (97). Moreover, macrophages near the vitreoretinal interface, mainly microglia, may act as biomarkers for inflammation (98). The emergence of diabetic complications, including microangiopathy, may be associated with a decrease in M2 polarization and a rise in the M1/M2 proportion. In pathological angiogenic ecology, retinal myeloid cells, especially macrophages/microglia, which are located close to endothelial cells in pathological angiogenic ecosystems, are highly glycolytic, express M1 and M2 markers more frequently, and produce more pro-inflammatory and pro-angiogenic cytokines (99). Angiogenic ability increases because of the interaction between macrophages and endothelial cells. Macrophages promote endothelial cell glycolysis and the lactate produced by glycolysis promotes macrophage activation, creating a positive feedback loop. Compared with normal retinas, patients with PDR have considerably more M1 and M2 macrophages in their fibrovascular membranes, and fibrovascular membranes development may be associated with M2 macrophages (100, 101).

The results mentioned in this section have led to the hypothesis that more than one type of polarized macrophage participate in retinal neovascularization. We can infer that in the early stages of DR, both pro-inflammatory (M1) and anti-inflammatory (M2) microglia are activated, and retinal adaptations increase to maintain their dynamic balance. Nevertheless, as the disease progresses, the number of M1 macrophages is maintained, whereas that of M2 macrophages gradually declines, favoring an inflammatory environment that causes retinal degeneration and loss of visual function.

## 5 Mechanisms of polarization in DR

Chronic hyperglycemia may cause gliosis by promoting the synthesis of advanced glycation end products (AGEs), which are macromolecules that are exposed to high blood glucose levels for a long period and become excessively glycated (102). Accumulated AGEs can trigger pro-inflammatory reactions via receptor (RAGE)-dependent or-independent mechanisms. In contrast to RAGE activation, which causes glial activation and the release of inflammatory cytokines, AGEs can cause endothelial cells to overexpress adhesion molecules (such as intercellular cell adhesion molecule-1) and subsequently activate leukocytes. Furthermore, oxidizing conditions and ROS generation connect retinal neuroinflammation with AGEs accumulation and create a positive feedback loop (103). Thus, inflammation and oxidative stress are the two main mechanisms involved in DR development and are closely related to macrophage polarization. Current research has mainly focused on the relationship among M1 polarization, oxidative stress, and inflammation. Both clinical and fundamental studies have shown that M2 polarization occurs in DR. Given that VEGF matters in retinal neovascularization and is a marker of M2 polarization, we also need to discuss the possible role of M2 polarization in pathological neovascularization in DR. We summarized the main signaling pathways related to polarization in **Table 2**.

**Table 2:** Signaling pathways related to polarization:.

Pathological process	Polarization phenotype	Signaling pathway	Authors
Inflammation	M1	NF-κB	Tang et al. (104) Fang et al. (105) Liu et al. (106) Chen et al. (107)
		TLR4/IRF5	Al-Rashed et al. (108)
		ROCK/JNK ROCK/ERK	Cheng et al. (109)
Oxidative stress	M1	ERK NF-κB	Khalid et al. (110) Yu et al. (111) Zhang et al. (112)
		TLR4/IRF5	Al-Rashed et al. (108)
Angiogenesis	M2	HIF-1α/VEGF/VEGFR2	Xu et al. (113) Liu et al. (114)
		histone acetyltransferase p300/spliced X-box binding protein 1/homocysteine-inducible endoplasmic reticulum protein with ubiquitin-like domain 1	Li et al. (115)
		CSF1/CSF-1R	Zhou et al. (116)
		prostaglandin E2/E-prostanoid 1 receptor/protein kinase C axis	Zhan et al. (117)

M1, conventionally activated macrophages; M2, alternatively activated macrophages; NF-κB, nuclear factor kappa-B; TLR, toll-like receptors; IRF, interferon regulatory factor; ROCK, Rho-associated coiled-coil containing protein kinase; JNK, c-Jun N-terminal kinase; ERK, extracellular regulated protein kinases; HIF, hypoxia inducible factor; VEGF, vascular endothelial growth factor; CSF, colony-stimulating factor.

### 5.1 Inflammation

Inflammation is one of the core pathological processes in DR. The expression of various inflammatory chemicals, including cytokines, chemokines, and growth factors is enhanced in the retina as a result of diabetes. The retina is already in a systemic pro-inflammatory environment before any clinical signs of DR manifest (118).

Macrophages/microglia located close to the vitreoretinal interface have been identified as possible indicators of inflammation in retinal vascular disease (98). M1 polarization and the generation of pro-inflammatory cytokines are both increased by activated NF-κB-related signaling pathways (104–106). Glucose fluctuations have been found to promote macrophage TLR4/IRF5 pathway activation, which in turn activate M1 macrophages and enhances the secretion of pro-inflammatory cytokines (108). A20, also called TNF-alpha-induced protein 3, negatively regulates M1 microglia. Glucose stimulation lowers ALKBH5 by m6A modification, resulting in reduced microglial A20 expression and promotion of M1 polarization in microglia (91). The mouse macrophage line Raw264.7 that was stimulated with high glucose levels showed that glucose primarily activates macrophages via the ROCK/JNK and ROCK/ERK pathways, promotes the conversion of the pro-inflammatory phenotype, and increases TNF-alpha expression (109). Aldose reductase, an enzyme linked to the pathophysiology of DR and strongly connected to LPS-induced M1 polarization, promotes microglial cell activation (119).

The communication between microglial cells has also been investigated. Exosomes produced from activated microglia boost microglial activation by sending polarized signals to M0 microglia via miR-155-5p (107). Inhibiting suppressor of cytokine signaling 1 and activating the NF-κB pathway, miR-155-5p eventually triggers an inflammatory cascade and intensifies the angiogenesis impact (107). In addition, studies have been conducted to determine how microglia interact with macroglia like Müller cells and astrocytes. Müller cells are susceptible to M1-polarized microglia and secrete more pro-inflammatory cytokines including IL-1beta, IL-6, and iNOS. Bidirectional signaling between the two cell types further boosts microglial activation and migration. Müller cells promote inflammatory responses throughout the retinal layer in chemotactic and sticky cell interactions, boosting microglia mobilization (120). Hyperglycemia affects retinal astrocyte proliferation, adhesion, and migration by producing inflammatory mediators (121). Translocator protein (TSPO), a microglial and astrocyte gliosis biomarker in cerebral deterioration, is acutely and selectively increased in the retinal microglia in an inflammatory environment. Likewise, astrocytes have increased levels of their endogenous ligand, diazepam-binding inhibitor (DBI). Microglial activation is adversely regulated by TSPO-mediated signaling via DBI-derived ligands (122). A network of well-coordinated astrocyte-microglial contacts is revealed by the inducibility and consequences of retinal DBI-TSPO signaling.

### 5.2 Oxidative stress

Free radicals are continually created in cells during metabolism under physiological conditions. Antioxidants are produced to neutralize these free radicals, ensuring a balance between them to support normal body functions. However, several metabolic issues can alter the ratio of free radicals to antioxidants, leading to an abundance of free radicals. Diabetes mellitus is a major metabolic disorder. Diabetes presents the retina with a double blow because it increases the formation of free radicals while simultaneously impairing their ability to be neutralized because of a dysfunctional antioxidant system (123). Cytosolic ROS damage the mitochondria, and mitochondrial damage enhances capillary cell death, which in turn causes diabetic retinopathy. This vicious cycle of ROS generation is further exacerbated by mitochondrial damage and continues to spread.

ERK and NF-κB signaling is activated by increased ROS levels and AGEs accumulation induced by hyperglycemia (110). Additionally, hyperglycemia stimulates HIF-1alpha-mediated VEGF production, which promotes neovascularization and serves a similar function by activating the ERK1/2-NF-κB signaling pathway in microglia (111, 112). The activation of TLR4/IRF5 signaling promotes M1 polarization and the production of matrix metalloproteinases (MMP)-9 at the same time (108). MMP-2 and MMP-9 are activated by increased ROS production, and with the aid of the heat shock protein 60, these MMPs penetrate the mitochondria. Subsequently, the mitochondrial membrane is damaged, which causes cytochrome c to leak into the cytoplasm and initiates apoptosis (124). Oxidative stress causes macrophages to polarize into the M1 form, which in turn promotes inflammation. Simultaneously, inflammatory cytokines are increased by oxidative stress and ROS may also be increased by inflammatory cytokines, forming a vicious cycle (125). Therefore, microglial polarization induces inflammation related to oxidative stress, which fosters DR emergence and progression.

Neurodegeneration and breakdown of the BRB are typical changes in the early DR stages. Before the development of clinical symptoms, the retinal neurons start decaying, and neuroretinal function is impaired prior to vascular lesions (25). Abnormal neural cell function, as well as pericyte and endothelial cell damage, are caused by the production of neurotoxic substances, including glutamate, oxidative stress, caspase-3, MMPs, and nitrous oxide (126). In the retinas of diabetic rats, activated microglia phagocytosed endothelial cells, increasing acellular capillaries and albumin leakage (127). Ding et al. (128) observed that LPS-activated M1 microglia may induce pericyte apoptosis in co-cultures. Increased levels of ROS secreted by M1 microglia may be the main reason for disruption of the inner BRB.

### 5.3 Angiogenesis

As mentioned in this section, fibrovascular membranes in DR formation may be associated with M2 macrophages. Recent research on DR has mostly focused on M1 polarization; studies of M2 polarization in DR are still lacking. Therefore, we summarized the role of M2 polarization in different retinal neovascular disorders to penetrate the similar potential roles and mechanisms of M2 polarization in DR.

Retinopathy of prematurity is a severe vasoproliferative disease caused by preterm birth or postnatal hyperoxia. OIR in animal models of retinopathy of prematurity are commonly used to study retinal neovascularization. Macrophages polarize toward the M2 phenotype throughout the course of retinal neovascularization, as evidenced by the fact that M1 macrophage levels change from considerably high levels at postnatal day 13 to nearly normal levels at postnatal day 21, whereas M2 macrophage levels rise from postnatal day 13 to day 21 (129). Moreover, M2 macrophages encircle emerging vessels and connect vascular sprouts. Co-culturing of human umbilical vein endothelial cells with M1- or M2-like macrophage supernatants has revealed that M2 macrophages are more effective in promoting proliferation and tube formation (130). M2 macrophage depletion by mannosylated clodronate liposomes inhibits pathological neovascularization, whereas intravitreally injecting M2 macrophages derived from the bone marrow has enhanced pathological neovascularization in OIR mice models (131). According to Wang et al. (132), increased retinal neovascularization is the outcome of M2 macrophages’ ability to attract and differentiate bone marrow-derived cells in response to stromal cell-derived factor-1 and VEGF. Furthermore, by stimulating signal transduction and activator of transcription 3, myeloid cells from circulation, particularly M2 macrophages, can travel through the lingual vascular system to the immature retina, which is ischemic and induces neovascularization (133).

Other investigations have looked into how M2 polarization influences neovascularization in AMD, which is a complicated, progressive, and degenerative disease influenced by several hereditary and environmental variables (134, 135). Two types of AMD manifest clinically: non-exudative senile macular degeneration, also called dry AMD, characterized by macular vitreous warts, pigmentary disorders, and map-like atrophy, and exudative senile macular degeneration, also called wet AMD, characterized mainly by choroidal neovascularization. The preponderance of M2 macrophages has been observed in both the mouse laser-induced choroidal neovascularization model and the atrial fluid of patients with wet AMD (136, 137). There may be a pathogenic change in the early AMD phases from pro-inflammatory M1 activity to protective M2 activity. M2 macrophages, in contrast, could serve a detrimental role by promoting fibrosis and angiogenesis. Macrophages stimulate VEGF production in laser- and AMD-associated choroidal neovascularization (138, 139). Activation of the HIF-1alpha/VEGF/VEGFR2 pathway has also been observed (113, 114). M2 macrophages are closely associated with VEGF production (132, 140). The histone acetyltransferase p300/spliced X-box binding protein 1/homocysteine-inducible endoplasmic reticulum protein with ubiquitin-like domain 1 (115), CSF1/CSF-1R (116) and prostaglandin E2/E-prostanoid 1 receptor/protein kinase C axis (117) can all, when hypoxic circumstances exist, stimulate M2 macrophage polarization, hence increase choroidal vascular endothelial cell proliferation, migration, and tube formation. M2 macrophages express leukotriene B4 (LTB4) receptor 1 (BLT1) which is drawn to the LTB to generate VEGF-A in a BLT1-dependent manner, inducing choroidal neovascularization formation (141). According to these investigations, M2 polarization is thought to be crucial for the growth of retinal neovascularization and to have a similar function in DR.

However, the specific role of M2 polarization in retinal neovascularization is still controversial. M2 macrophages are considered to play a protective function in tissue repair and remodeling (142). In a study of phase changes following OIR, in the late phase after postnatal day 17, IL-4/STAT6/PPAR-gamma signaling activity is heightened, activating M2 macrophages and peaks at postnatal day 20, causing the reduction of inflammation and the naturally occurring regression of neovascularization clusters (143). Due to a change in macrophage polarization towards an M2 phenotype, the OIR model that administered IL-17A neutralizing antibody had noticeably less retinal neovascularization than normal mice (144). Injecting umbilical cord blood-derived CD14^(+)^ cells into OIR eyes stabilizes retinal ischemia-damaged vessels after subdivision into M2 macrophages (145). By controlling the inflammatory response, promoting tissue repair, accelerating nutritional recovery, enhancing the clearance of apoptotic cellular debris, and becoming tolerant instead of autoimmune, M2 macrophages aid in the normalization of the retinal vasculature. These effects are reinforced by the fact that M2 macrophages participate in downstream vascular anastomosis of VEGF-activated endothelial tip cells (146).

**Figure 2:**
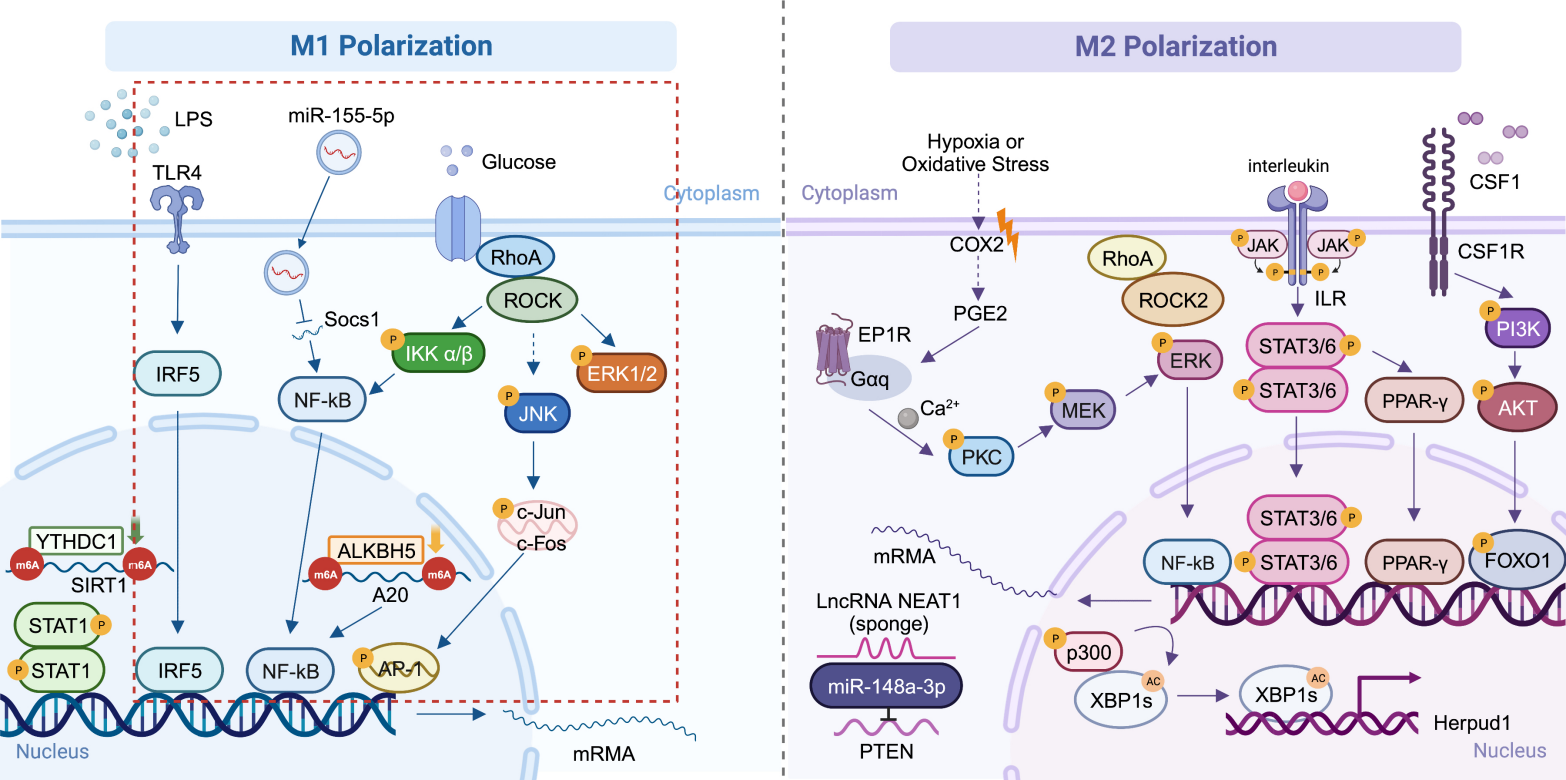
Mechanisms of macrophage polarization in ocular diseases. The main DR-related mechanisms are circled in red (91, 108, 109, 115–117, 134, 147, 148). Created with BioRender.com. M1, conventionally activated macrophages; M2, alternatively activated macrophages; DR, diabetic retinopathy.

In summary, microglial polarization affects inflammation and oxidative stress caused by sustained tissue stress caused by hyperglycemia in DR, during which M1 macrophages play a critical role. The two processes interact with each other and promote the pathological alterations in DR. Pericyte loss occurs followed by endothelial cell injury due to chronic inflammation, eventually the BRB is destroyed and neoangiogenesis arises. M1 polarization has been the main subject of the majority of recent investigations on microglia polarization in DR. Other ocular neovascular disorders have been found to be related to both the pro-angiogenic and protective effects of M2 macrophages. Since DR is a typical retinal neovascularization disease caused by increased VEGF levels, the specific role of M2 macrophages in the pathogenesis of DR needs further study. Additionally, we have integrated some currently recognized polarization pathways linked to ophthalmic diseases (**Figure 2**). Whether the same mechanisms that cause polarization in other diseases also play a role in DR requires further investigation.

## 6 New strategies for DR treatment targeting polarization

Considering the dominant role of M1 polarization in DR, current therapeutic approaches mainly focus on inhibiting the pro-inflammatory response to M1 polarization and promoting the switch of M1 to M2 polarization. Studies about the treatments related to polarization were summarized in **Table 3**.

**Table 3:** Treatments associated with polarization:.

Treatment	Detailed name	Administration	Pharmacological effect	Authors
medication	Melatonin	Intravitreal injection	Inhibit NF-κB signaling	Tang et al. (104)
	Pyrrolidinedithiocarbamate			Sui et al. (149)
	Asiatic acid	Oral administration		Fang et al. (105)
	Cyanidin 3-O-glucoside			Zhao et al. (150)
	Ferulic acid			Sun et al. (151)
	(S_S_,1R)-1-docecylsulfiny-5N,6O-oxomethylidenenojirimycin ((Ss)-DS-ONJ)	*In vitro* experiment	Boost M2 responses	Cano-Cano et al. (152)
	1-dodecylsulfonyl-5N, 6O-oxomethylidenenojirimycin (DSO_2_-ONJ)			Alcalde-Estévez et al. (153)
	sp_2_-iminosugar dodecylsulfoxide derivative R-DS-ONJ			Arroba et al. (81)
	Anti-VEGF agent	Intravitreal injection	Regulate DBI-TSPO signaling	Gao et al. (154)
	Sorbinil, β-glucogallin	*In vitro* experiment	Inhibit aldose reductase	Chang et al. (119)
	Sorbinil	Intraperitoneal injection		Chang et al. (155) Rao et al. (156)
	Sorbinil	*In vitro* experiment		Huang et al. (157)
Stem cell therapy	Human umbilical cord blood	Intravitreal injection	Phenotypic transition from M1 to M2	Marchetti et al. (145) El Kasmi et al. (158) Panopoulos et al. (159) Ritter et al. (160) Xu et al. (161)
	Mesenchymal stem cells	*In vitro* experiment		An et al. (162) Jaimes et al. (163) Teixeira-Pinheiro et al. (164) Honda et al. (165)
	Bone marrow-derived precursor cells			Medina et al. (166) Nakagawa et al. (167)

M1, conventionally activated macrophages; M2, alternatively activated macrophages; NF-κB, nuclear factor kappa-B; DBI, diazepam-binding inhibitor; TSPO, translocator protein.

### 6.1 Signaling inhibition related therapy

We can draw the conclusion that NF-κB signaling pathway really matters in M1-type polarization from the previously mentioned mechanisms. In order to effectively treat DR by targeting polarization, more attention is given to NF-κB signaling inhibitors. Melatonin (104) and pyrrolidinedithiocarbamate (149) injection into the vitreous cavity and Asiatic acid (105), cyanidin 3-O-glucoside (150) and ferulic acid (151) oral administration were reported to decrease NF-κB signaling, reduce inflammatory lesion and M1 polarization, and promote M2 polarization in the retina of rats with DR, therefore minimizing the lesions. They may simultaneously maintain BRB integrity by controlling the expression of proteins associated with barriers. The sp^2^-iminosugar glycolipid (sp^2^-IGL) family, (S_S_,1R)-1-docecylsulfiny-5N,6O-oxomethylidenenojirimycin ((Ss)-DS-ONJ), increases the expression of heme oxygenase-1 and IL-10 in endotoxin-stimulated microglia, thereby exerting anti-inflammatory effects by inducing arginase-1 to boost M2 responses (152). Other members of the sp^2^-IGL family, such as 1-dodecylsulfonyl-5N, 6O-oxomethylidenenojirimycin (DSO_2_-ONJ) (153) and the sp_2_-iminosugar dodecylsulfoxide derivative R-DS-ONJ (81) have also been shown to perform a similar function, alleviating DR. In addition, DBI-TSPO signaling in the retina is regulated by VEGF, which can alleviate the severity of inflammation, promote the production of neurotrophic factors, polarize M2 macrophages, and relieve neovascular retinopathy (154). Considering the role of aldose reductase in M1 polarization, pharmacological inhibition of aldose reductase could relieve M1 polarization induced by LPS, decelerate optic nerve degeneration, and decrease the capacity of activated microglia to induce apoptosis in retinal pigment epithelium cells, thus protecting the integrity of the BRB (119, 155–157).

### 6.2 Stem cell related therapy

Macrophages/microglial cells differentiate and develop from bone marrow hematopoietic cells. Based on different macrophage phenotypes and the conversion of phenotypes, a rising variety of researches have concentrated on the underlying value of bone marrow stem cell transplantation for the treatment of intravenous pathological neovascularization and retinal neurodegenerative diseases (166, 168–170). Numerous studies have demonstrated that intravenously injected stem cells or local transplantation can enhance function in ischemic regions (171–173). Stem cells can develop into endothelial (174, 175), neuronal (176, 177), and mature myeloid cells (such as, monocytes, macrophages, and dendritic cells) *in vitro*. According to recent studies, distinct tissue microenvironments subject myeloid cells to various stimuli and determine whether they differentiate into inflammatory macrophages (M1 and M2), endothelial cells, or dendritic cells (41, 178–180). Therefore, various myeloid cell subpopulations may have opposing effects on angiogenesis; one population may support angiogenesis in tumors (181), whereas another may suppress tip cell numbers and vascular sprouting (182).

Mesenchymal stem cells (MSCs) are found in various tissues and can differentiate in several directions. MSCs are a potential therapy for inflammatory illnesses because a significant body of research has shown that they are potent immunomodulators. They often influence microglial polarization via paracrine actions (183). MSCs inhibit NF-κB activation and excretion of TNF-alpha stimulated protein 6 in human umbilical vein endothelial cells co-stimulated by hyperglycemia and megadose of palmitic acid, thereby suppressing inflammation (162). After co-culturing with MSC-derived microvesicles, increased levels of M2 markers and fewer LPS-induced pro-inflammatory cytokines were detected in BV-2 cells, indicating their role in the phenotypic transition from M1 to M2 (163). To observe the role of MSCs in the retina, MSCs obtained from human umbilical cords were co-cultured with complete adult rat retinal explants and kept apart using a transwell membrane (164). Due to a decline in M1 markers and a raise in M2 markers, paracrine signaling of hMSC altered the microglial phenotype and the production of anti-inflammatory proteins in the retina (164). In neurological studies, MSCs have been found to induce M2 polarization in macrophages and facilitate their migration into MSCs *in vitro* (165). Therefore, it is conceivable that injecting MSCs into the vitreous cavity may be a potential therapeutic approach for treating DR since it may increase the number of microglia with anti-inflammatory phenotypes and help preserve the retina.

According to Marchetti et al. (145) injecting the human umbilical cord blood into the vitreous cavity has a significant curative effect, creating abundant myeloid progenitor CD14^(+)^ cells. CD14^(+)^ cells can stop abnormal neovascularization and polarize into M2 macrophages. For the sake of managing cell proliferation, differentiation, and the resolution of inflammation, CD14^(+)^ cells develop into a macrophage oriented M2 type, inducing the production of genes participating in cellular signals such as IFI6, IFI1631, BCL2A1, AKT1, NF-κB1, and TGF-beta1. They not only ease oxidative stress, apoptosis, inflammation, and angiogenesis (145, 158, 159) but also stabilize the retinal vasculature (160, 161). Furthermore, transcriptome sequencing has validated the biological functions of CD14^(+)^ cells.

Nakagawa et al. (167) also confirmed that bone marrow-derived precursor cells can migrate to the retinal vascular network, differentiate into glial cells, and promote the steady state of the blood vessels of the retina, which plays an essential role in retinal vascular reconstruction and stabilization. Therefore, they hypothesized that this protective effect may be intimately linked to macrophage/microglia polarization. To determine whether myeloid angiogenic cells exhibit pro-angiogenic characteristics, Medina et al. (166) employed a 3D Matrigel tube formation assay and discovered that myeloid angiogenic cells might cause endothelial tube development *in vitro* in a paracrine manner. Additional research focusing on M1/M2 markers and pro-angiogenic genes revealed that myeloid angiogenic cells significantly expressed M2 macrophage markers, such as IL-10, transforming growth factor beta 2, CD163, macrophage scavenger receptor 1, and mannose receptor C type 1. However, pro-inflammatory M1 macrophage markers, such as TNF, IL-1, IL-6, IL-12, and cytochrome c oxidase subunit II were almost undetected. The pro-angiogenic genes VEGF-beta, connective tissue growth factor, platelet-derived growth factor beta polypeptide, neuropilin 1, and MMP-9 were also strongly expressed. This study demonstrated that M1 macrophage markers were downregulated, whereas pro-angiogenic genes and M2 macrophage markers were highly upregulated.

Overall, stem cells are a promising treatment for DR because of their pluripotency. More critically, DR development may be significantly slowed by the promotion of stem cell differentiation into M2 macrophages with anti-inflammatory capabilities.

## 7 Discussion

As important immunoregulatory cells, the extensive distribution and function of macrophages and microglia have become a research hotspot. Recently, researches have focused more on polarization and differentiation in cancer, cardiovascular diseases, obesity, and neovascularization. In ocular diseases, macrophage/microglia polarization is associated with the onset of illness, especially DR. The inflammatory stage appears to be dominated by M1 polarization, which is also correlated with the beginning of inflammation and the onset of early tissue damage. Disease relief and tissue healing are highly correlated with M2 polarization. However, it is also crucial to remember that increased M2 polarization may play a role in several aberrant healing processes, including fibrosis and angiogenesis. Phenotype conversion not only reflects the tissue microenvironment inflammatory state but also suggests the severity of pathological neovascularization, which affects the outcome and prognosis of the disease.

The present review demonstrated that early DR, even at the preclinical stage, is accompanied by both M1 and M2 polarization, whereas only M1 polarization is effective in the latter phases. The classic change in the latter phase of DR (PDR) is the secretion of large quantities of VEGF, which results in retinal neovascularization. However, M2 polarization is often considered to result in excessive VEGF release. Therefore, further research is needed to explore whether M2 polarization continues to play a significant role in the later DR stages so that more precise treatment can be realized. Moreover, the target of current treatments is to convert M1 polarization to M2 polarization. It is still doubtful whether such medication will boost VEGF output and aggravate the lesion, despite the possibility that it will reduce inflammation. Inflammation and neovascularization signaling pathways often crosstalk, and persistent chronic inflammation can result in neovascularization. The M2 macrophages are mostly anti-inflammatory and pro-neovascular cells. If M2 macrophages encourage the formation of structurally and functionally intact neovascularization to replace pathogenic neovascularization in DR, this may represent a potential therapeutic strategy. A better approach is to focus more on the transformation of resting M0 macrophages, which should be further investigated. Stem cell therapy deserves more attention as a personalized treatment. Depending on its pluripotency, they may differentiate into various macrophage states, including M1, M2, and even quiescent M0, thereby opening up more treatment options. Depending on an individual patient’s retinal microenvironment, different measures can be implemented on a case-by-case basis, thus maximizing personalization.

Given that the current research is not in-depth, further exploration of the internal mechanisms of macrophage/microglial polarization is important for understanding ocular diseases. The “double” polarization of macrophages/microglia remains controversial, its plasticity and diversity need to be further studied and assessed. It is still being determined whether some functional categories reflect different populations because macrophage polarization characteristics are not unique. It is also possible to reverse polarization imbalances, thus intervening in disease development. These findings will likely provide new therapeutic strategies for DR and bring up new therapy options for ocular disorders.

## Author contributions

YY: Writing – original draft, Visualization. JL: Writing – original draft, Writing – review & editing. YZ: Writing – original draft, Writing – review & editing. SW: Writing – original draft. ZZ: Writing – original draft. QJ: Writing – review & editing, Supervision. KL: Writing – review & editing, Supervision, Funding acquisition.

## Glossary

**Table:**

M1	Classically activated macrophages
M2	Alternatively activated macrophages
DR	Diabetic retinopathy
OIR	Oxygen-induced retinopathy
AMD	Age-related macular degeneration
VEGF	Vascular endothelial growth factor
NPDR	Non-proliferative diabetic retinopathy
PDR	Proliferative diabetic retinopathy
CSF	Colony stimulating factor
IFN	Interferon
TNF	Tumor necrosis factor
LPS	Lipopolysaccharide
IL	Interleukin
iNOS	Inducible nitric oxide synthase
ROI	Reactive oxygen intermediates
TGF	Transforming growth factor
TLR	Toll-like receptor
TAM	Tumor-associated macrophage
NFL	Nerve fiber layer
GCL	Ganglion cell layer
IPL	Inner plexiform layer
OPL	Outer plexiform layer
BRB	Blood-retina barrier
AGEs	Advanced glycation end products
ROS	Reactive oxygen species
TSPO	Translocator protein
DBI	Diazepam-binding inhibitor
MMP	Matrix metalloproteinases
LTB4	Leukotriene B4
BLT1	Leukotriene B4 receptor 1
MSCs	Mesenchymal stem cells
NF-κB	Nuclear factor kappa-B
IRF	Interferon regulatory factor
ROCK	Rho-associated coiled-coil containing protein kinase
JNK	c-Jun N-terminal kinase
ERK	Extracellular regulated protein kinases
HIF	Hypoxia inducible factor
